# A Plea for Conservative Minor Gynecology

**Published:** 1891-06

**Authors:** R. R. Kime

**Affiliations:** Atlanta, Ga.


### A PLEA FOR CONSERVATIVE MINOR GYNECOLOGY.

BY R. R. KIME, M. D., ATLANTA, GA.

Shall the uterus be curetted? Is the curette the direct cause of pelvic complications requiring abdominal section, and shall its use be abandoned? Are questions that confront us for consideration when some of our eminent gynecologists are attributing many abdominal sections to minor gynecological work, classing as potent factors for evil the curette in the same category as the uterine sound. To quote from Dr. Joseph Price on the use of the sound, "If used at all, it should be in the hands of those with whom its application, by reason of their skill, will be exceptional, not usual, and the rule should be, that in the hands of the non-expert it should be forbidden, . . . . Along with the sound may be placed the curette in the same category."

This certainly is an extreme view of the use of the curette, and while an improper, injudicious use of the curette may often cause pelvic difficulties requiring abdominal section, it certainly is often a very potent factor, properly used, in preventing those conditions requiring abdominal section. Because serious results follow the injudicious use of the curette, it should not constitute an edict to consign its use to oblivion any more than abdominal section should be consigned to oblivion because of its abuse and the disastrous results following some operations.

If it can be established that the curette has accomplished good, relieved suffering woman, prevented more serious complications, then its abuse should be condemned and not its use.

In considering this subject we will necessarily have to enter to some extent the domain of obstetrics, because the complications incident to labor, abortion and miscarriage often bear a direct relation to gynecic surgery. It is not the object of this article to discuss antiseptic or aseptic obstetrics, but to deal, so far as the application of the curette is concerned, with some of the complications following labor, abortion and miscarriage. It is the teaching of surgery where we have foreign, diseased, broken-down tissues to scoop out, scrape out, remove, cleanse cavity, drain and dress antiseptically.

It would certainly be rational to apply same principles to the uterus where it contains foreign substances and diseased tissues, broken-down decomposing substances, or any structures keeping up continuous waste, sapping the vital powers of patient.

In septic infection or putrid intoxication, where uterus contains decomposing germ producing material, constantly being absorbed, menacing the life of the patient, wash out cavity of uterus, disinfect it — curette — again wash out, disinfect, drain and dress aseptically. If such procedures are instituted while the foe is yet confined to the uterus, before system is seriously affected by absorption or serious lesions have developed in other pelvic organs, I can well understand how abdominal section can be prevented rather than caused. By curetting thus performed, at the proper time, I think it is possible often to prevent subinvolution, pelvic peritonitis (pelvic cellulitis?), salpingitis, ovaritis and pyosalpinx, etc., thereby lessening the income of the abdominal surgeon.

In abortion and miscarriage there is certainly a wide range of usefulness for the curette. In those cases where nature fails to perform her work properly, the fœtal structures passing off leaving maternal structures in uterine cavity to produce hemorrhage, decompose, causing more serious lesions when the condition of the uterus is such as to prevent the efficient removal of its contents by finger or hand, then I would say curette.

During the third month of pregnancy, when the ovular decidua is rapidly atrophying, while the decidua serotina or placental decidua is as rapidly developing into the maternal portion of placenta, we are so likely to have those cases where the fœtal structures pass off, leaving the newly formed maternal structures behind, causing hemorrhage that saps the vital powers of the patient, rendering her more susceptible to the deleterious influences occasioned by the decomposition of the retained structures. Allowing such a condition of affairs to continue is often the cause of many of the diseases the gynecologist is called to treat and the abdominal surgeon to relieve.

By the judicious use of the curette in these cases many complications can be averted, life saved and the use of the knife prevented.

To illustrate more clearly I report :

Case I.—Mrs. S——, multipara, missed two flows. Complains of pains in bowels and pelvic region, nausea, headache, coated tongue, weak, debilitated.

Prescribed tonics, uterine sedatives, rest and nourishment. Five days later found her wasting freely, abortion unavoidable, fœtal structures passed off, unable to remove maternal structures manually on account of pain. Used curette forceps and currette, irrigating and disinfecting uterine cavity. Rapid recovery without further wasting, although it had existed for several days, as well as the elevation of temperature.

II.—Called in consultation to see Mrs. L——. Fœtal structures having passed off about one week previous, attending physician had been unable to remove maternal structures, although an able physician of years of experience. There being elevation of temperature, evidences of decomposition and absorption. I at once curetted with the usual precautions. Temperature subsided, discharges checked, rapid recovery.

III.—Called to see Mrs. R——, in consultation. Had been flowing some ten or twelve days, attending physician having failed to remove uterine contents. Patient refusing to have curette used, after I had tried to clear uterine cavity with finger, I returned home. After five days of continually getting worse, I

was called again. Curetted with the usual result, rapid recovery.

IV.—Mrs. M——, primipara, supposed three months pregnant; thrown from a mule; was called a few days later. Found her with elevation of temperature, excessive nausea, furred tongue, red on edges, tympanites, constipation, pelvic tenderness but no exudate as far as able to detect.

Prescribed rest, opiate viburnum, remedies to quiet nausea, to be followed by carthartic. Patient grew worse, fœtal structures passing off some seven or eight days later, placental structures retained. As I could not remove them without instruments, gave usual treatment for two days then curetted. Patient gradually improved, inflammatory symptoms subsiding, recovered.

V.—Was called to see Mrs. N——, multipara, in third month of pregnancy, suffering with pelvic inflammation, with exudate surrounding the whole of uterus; four days later general peritonitis developed; five days laterfœtal structures passed off; patient in a critical condition, temperature 97 degrees Fahrenheit, pulse 135, weak. There being considerable hemorrhage tamponed for fourteen hours, then tried again to remove uterine contents with finger; failing, proceeded to curette. Patient then had a temperature of 96½ degrees Fahrenheit, pulse 130, discharges offensive. Found uterus fixed immovably by inflammatory exudate surrounding it, with a constriction in cavity 2½ inches from os, 1½ inches from fundus; placental structure partly above and partly below constriction, which could be dilated easily with a Goodell-Elinger dilator, but rapidly contracted as soon as instrument was removed. Succeeded comparatively well in emptying uterus, disinfecting thoroughly. Temperature soon reached normal, pulse came down, patient improved rapidly for a few days, when a change of weather came, rain blew in above and below door, wetting carpet, etc.; patient "took cold," began coughing, rekindled pelvic inflamation, which soon subsided under appropriate treatment, and patient recovered.

Other cases might be presented, but these certainly illustrate that the curette benefits some cases, and to my mind prevents graver complications.

It may be claimed this curetting is all unnecessary, that a physician should be able to manage all such cases without instruments or that nature can take care of them. I would reply, nature often does not, and the physician who fails to relieve his patient, if need be, with curette and prevent the ebbing away of that "which is the life thereof" and the development of those graver complications and sometimes death, is culpably negligent of his duty.

As to my not being able at all times to remove placental structures from uterine cavity *without instruments*, I do not feel that it is entirely a want of skill, even be it so. I know I have at my command means by which I can do such work more efficiently, with less pain, without anæsthetic or assistant, and with less risk to patient than to allow them to remain. I am also inclined to think that a pelvic inflammation in the conditions under consideration is not always a barrier to the use of the curette. Nay, I believe it is sometimes prevented, if not relieved, by the proper use of the curette.

Certainly I would not claim that where pyosalpinx, ovarian or pelvic abscess exists that curetting the uterus would relieve them, yet I would not fear to curette the uterus, with proper precautions, where metritis, salpingitis, ovaritis, and even where a pyosalpinx existed on one side previous to the pregnancy, if the uterus contained placental tissues keeping up a continual waste, decomposing, and endangering the life of the patient.

A word as to mode of curetting. Put patient on back, using a Hale-Leonardo-Wackerhagen speculum, a variety that expands vulval orifice, allowing space for use of instruments. If patient is in Sims' position use an Erich-Cleveland or some self-retaining speculum, dilating if necessary with steel dilators, never using any form of tents. Irrigate with mercuric chloride 1 in 2,000 to 4,000 or 6,000; curette with sharp or dull curette as the case demands. Again irrigate, being certain to give free exit to all fluids injected. If need be hold dilator in cervix while irrigating. If the stronger solutions are used, follow with sterilized water to prevent mercurial poisoning, then apply to endometrium pure carbolic acid, iodized phenol, or inject twenty to thirty min. of Churchill's tincture iodine. Remove excess with absorbent cotton, drying vaginal canal. Dust cervix thoroughly with iodoform or boracic acid. Introduce a pledget of borated cotton, with string attached, to be removed in ten or twelve hours, following with copious hot water vaginal irrigation.

I have thus carried out this procedure a number of times, without assistant or anæsthetic, with less pain to patient than removal by any other means, and, I dare say, more efficiently than it can be done with finger and with more satisfactory results.

The operation, performed as above indicated, with aseptic instruments and aseptic operator, will not cause, but prevent, many abdominal sections. Even where salpingitis, ovaritis or pelvic inflammations exist as a result of septic absorption from uterine contents, they will not be aggravated, but lessened, by such a procedure, often preventing abscess formation requiring grave procedures.

